# Royal Hampshire County Hospital

**Published:** 1909-11-06

**Authors:** J. Charles Warner

**Affiliations:** Chairman of and on behalf of the Committee of Management. Winchester


### ROYAL HAMPSHIRE COUNTY HOSPITAL.

Dear Sir,—Sir Henry Burdett, K.C.B., K.C.V.O., made a visit of about two to three hours' duration to the hospital in August last, and apparently from that brief visit, and possibly from a perusal of the last annual report of the Charity, he has published in your journal of October 16 an interesting report of this hospital. We appreciate most fully and concur in the just and generous remarks upon our resident staff, we extend the same appreciation to our honorary and non-resident medical staff. We venture to think the "hospital spirit," to which he refers in such high terms, has been the growth of many years, created and fostered by the medical staff of an earlier day, and possibly by the Governors and Committee of Management through whose care and energy the hospital is just able to face its financial responsibilities.

We feel, however, that the report in some other respects is not fair to the charity, which is supported by voluntary contributions and managed by a voluntary Committee and an Honorary Secretary. We repudiate most distinctly the statement "that the interior is relatively dirty and unattractive"; what is attractive is a matter of taste, but "dirty" is not a word to apply to the interior of a hospital without the clearest proof of its truth. The interior in parts may be dingy or discoloured, but it is not dirty.

To pass, however, to the main criticism, it is summed up in the words "the enforcement of economy with so rigid a hand as to reduce it to a fault." Perhaps Sir Henry Burdett is unaware that in order to make the hospital fit for the great work it performs the Governors, through the Committee, have expended since the year 1897, £23,128 for the following purposes, and on the best expert advice that could be obtained—namely, a new system of heating, a complete new modern system of drainage, the rearrangement and improvement of the out-patients department and the dispensary, a new steam laundry, outside staircases for escape in case of fire, installation of electric light and power, a new operating theatre, and the new nursing block of which he speaks so well. Part of this expenditure was supplied by special contributions, but the Committee were compelled to sell investments to the amount of £12,344 to provide the balance, and the income derived from this sum has therefore been lost to the hospital. These constructive works hardly justify the criticism of want of active administration.

Our annual income, with the exception of the past year, when it was increased by special donations of £1,300, has for many years proved utterly insufficient to provide for the ordinary annual expenditure. The excess of annual expenditure over annual income of recent years has been as follows:—1901, £2,887 15s. 1d.; 1902, £407 10s. 8d.; 1903, £741 17s. 9d.; 1904, £1,582 11s. 2d.; 1905, £449 16s. 10d.; 1906, £795 4s. 4d.; 1907, £1,107 16s.; 1908, £1,493 0s. 10d. Thus our financial crises have been frequent, and unless generous friends in the City and county had come to our aid last year with these large and unexpected donations we must have closed one, if not two, wards. To show the increase of the usefulness of the Charity, the number of in-patients treated in the year 1897 was 723, and in the year 1908 1,200, and the number of operations performed in the year 1908 was 822.

No request has been made to the committee by the matron or resident medical staff for additional help nor have suggestions been received from them of overwork. They are most loyal to the Committee and Charity and we think it would have been better if this question had been first submitted to the Committee of Management before any public criticism was made. This is not the time or place to discuss the terms upon which our visiting nurses are engaged. We think that on a closer examination of the circumstances under which our visiting nurses are engaged Sir Henry Burdett will find that there is no unfairness in our treatment of them, but that they receive a just and proper payment for their services.

With regard to the letters of admission, there is much to be said in favour of their abolition; but the Committee feel that they are better judges of local conditions and so retain them. The present scale was fixed many years ago after an exhaustive inquiry, but it may be well now, under altered conditions, to reconsider this scale. The financial policy which the Committee adopt is that of endeavouring to avoid debt, and at the same time to expend the money at their disposal in the best possible way with the readily-given assistance of the whole staff.

J. Charles Warner,
Chairman of and on behalf of the
Committee of Management.

Winchester, November 2, 1909.

[*** We have pleasure in publishing Mr. Warner's letter. We note with satisfaction that in certain directions indicated by Mr. Warner the Committee has endeavoured to meet modern requirements. Mr. Warner's letter, however, confirms and enforces the points urged by Sir Henry Burdett in his report. It admits that the interior is "dingy and discoloured," or, as Sir Henry puts it, "dirty and unattractive" in appearance. We know from representations made to us since the report was published that influential governors agree with that report, and consider a change in the present policy of the Committee essential to the best interests of this hospital. It is the duty of every Committee to realise when the staff is overworked and to provide against this fault. Men and women of the highest character will not complain when overworked in a hospital to which they are devoting all their energies. What the governors and the public want to know is, will the Committee act promptly, or does it intend to do nothing? Will it, for instance, (1) redecorate the interior of the hospital and make it smart and cleanly in appearance? (2) bring the staff into line with the best-managed hospitals of the day by (*a*) appointing an assistant to the lady superintendent; and (*b*) providing an extra medical resident? (3) change the present system "of starvation in little things"? (4) prove that it is animated by the hospital spirit by using energy and vigour in the prompt removal of the defects which at present mar much which is so excellent and exemplary? (5) face the financial position by declining longer to give £5 17s. 6d. in actual value to each subscriber of £2 2s. per annum? To continue such a system is neither philanthropy nor business; to change it by making it both should put the hospital in funds and prevent any excess of expenditure over income in future years. Finally, will it (6) recognise, like other well-administered hospitals, that nurses are entitled to the larger share of their earnings, and that no properly managed modern hospital can farm nurses out at a profit without losing prestige and reputation?—Ed. The Hospital.]

